# **Phytochemical based on nanoparticles for neurodegenerative alzheimer disease management: Update review**

**DOI:** 10.1186/s11671-025-04356-x

**Published:** 2025-10-09

**Authors:** Basma Youssef, Ehab A. Ibrahim, Said S. Moselhy, Shaimaa ElShebiney, Walaa K. Elabd

**Affiliations:** 1 Biochemistry Department, Faculty of Science, Ain Shams University, Cairo, Egypt; 2 Department of Narcotics, Ergogenics and Poisons National Research Centre, Cairo, Egypt

**Keywords:** Dementia, Blood-brain barrier, Alzheimer's disease, Nano-medicine

## Abstract

Alzheimer’s disease (AD) is one of the most common chronic neurodegenerative diseases and dementia, with about 46 million cases worldwide and going to become tripled by 2050. It is characterized by formation and aggregation of amyloid-*β* plaques, tau tangles, and inflammatory mediators. The treatment protocol poses challenging obstacles particularly, effectiveness drug delivery to the brain. However, the available therapies with low potency and blood-brain barrier (BBB) are most challenges for developing novel treatments. New delivery systems that interact with biological systems at the molecular level, such as nanotechnology can overcome these problems and open new therapeutic avenues. Nanoparticles showed different applications in medicine due to its bioavailability, transport and low toxicity. This review explored the therapeutic potential of natural phytochemical nanomedicine that important in AD treatment through improving drug delivery system across BBB, increasing bioavailability and minimizing neurotoxicity.

**Graphical abstract:**

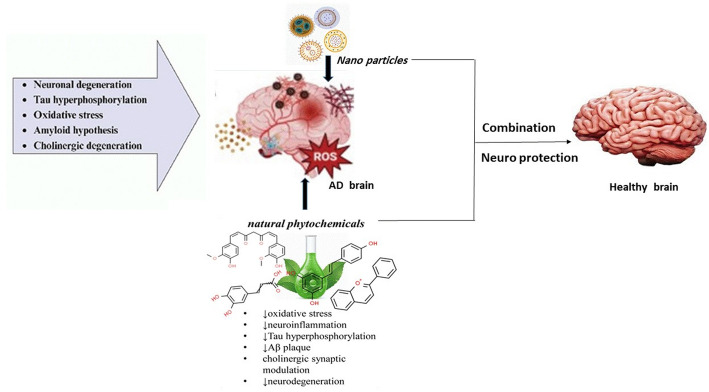

**Supplementary Information:**

The online version contains supplementary material available at 10.1186/s11671-025-04356-x.

## Introduction

Neurodegenerative disorders (NDs) are characterized by a premediated loss of neurons, these include progressive neuropsychiatric conditions, such as Alzheimer’s disease (AD), Multiple sclerosis (MS), Parkinson’s disease (PD), and various other NDs. Alzheimer disease (AD) is one of the most prevalent chronic NDs [42] that is characterized by cognitive impairment, disorientation, and memory loss [149]. AD is the most frequent cause of dementia, hitting 50 million people and predicted to reach 150 million by 2050 [129]. NDs include the progressive loss of neurons, resulting in cognitive, motor, and various neurological deficits [4]. Nowadays, conventional AD treatments seek to enhance symptoms but lack specificity due to the blood brain barrier (BBB) resulting in systemic side effects [25]. The usage of new therapeutic nano-delivery techniques may help to overcome the BBB challenge. Nano medicine in one aspect considers combining nanotechnology and medicine to deliver therapeutic drugs to target regions [60].These nanoscale structures make it easier for drugs to cross the BBB, enhancing their pharmacokinetics, pharmacodynamics, and bioavailability. Polymeric NPs, dendrimers, and lipid-based NPs are examples of such nano carriers[103]. Several reports discussed this application in various disorders and outlined superiority to conventional therapies in terms of efficiency and targeting [138]. AD is considered as one of the most neurological diseases that threaten mental health. Nowadays treatments are far from therapeutic need and have several side effects. Traditional drugs used to treat AD may fail to reach the cells that require treatment. Furthermore, Natural plant derived medicines have received a great deal of attention. Their clinical utility is limited as a number of shortcomings. Furthermore, Nanotechnology is critical for treating a variety of diseases, including AD. The current review identified the recent applications of phytochemical based nano medicine as a potent therapeutic tool for AD, highlights how natural substances with anti-inflammatory, antioxidant, and neuroprotective properties can be effectively delivered through nanocarriers bypassing BBB via nanocarriers. This approach, which prioritizes brain targeting, bioavailability, and multi-modal action, offers a potential alternative to traditional synthetic drug treatments, also it gathers relevant information concerning the barriers in drug design for AD and the clinical use of NPs in the development of a new formulation for AD treatment.

## Materials

Interpretation and analysis of updated information collected from databases web sites such as PubMed, NIH, Scopus and Web of Science.

### Alzheimer's disease (AD)

Neurodegenerative disorders cause subsequent symptoms including memory loss and impaired coordination, as well as a complete inability to operate as a typical, healthy person [121]. The most common neurodegenerative disorders are Parkinson's disease (PD), AD, amyotrophic lateral sclerosis and Huntington’s disease [31]. AD is the most prevalent cause of dementia globally, accounting for 60–70% of cases. An increasing number of researches indicate that a combination of brain disorders, as AD, accounts for the majority of dementia cases in the elderly [38]. AD is associated with atrophy in the hippocampus and cerebral cortical regions, especially in the frontal–temporal horn, resulting in memory and spatial learning impairments [71]. AD patients can be identified by altered neurotransmitter expression, decline in neutrophil counts, *Aβ*-protein deposits (amyloid/senile plaques), which can trigger dendritic spine loss, and, in the latter stages of the disease, extensive neuronal death and brain atrophy according to various studies [139].

### AD pathophysiology

The pathological features of AD include the gradual degradation of neurons and synapses, along with the formation of diagnostic amyloid plaques composed of fibril β-amyloid peptide aggregates and neurofibrillary tangles formed of hyper phosphorylated tau protein filaments. Recent research has demonstrated the importance of tau molecules and soluble-amyloid oligomers, despite the previous belief that plaques and tangles were the primary mediators of neurotoxicity in AD [61]. AD has a complicated pathophysiology that includes several factors that can affect neuronal function and communication, such as inflammatory processes, mitochondrial dysfunction, synapse dysfunction, Aβ and tau protein,and cholinergic signaling, particularly acetylcholinesterase [87].

### Molecular mechanisms & the hypothesis of β-amyloid aggregation in AD

The Aβ plaques, which are insoluble peptides, are formed when amyloid precursor proteins (APP) APP segmentation goes incorrectly [18].The precise role of these plaques play in the development of the illness is yet unknown. Three enzymes, α-secretase, β-secretase, and γ-secretase, catalyze the metabolism of APP to produce several metabolites. The products of β-secretase are usually metabolized by γ-secretase, resulting in the formation of soluble peptides with 40 amino acids (Ayaz et al. [10]). An abnormal APP cleavage caused by a mutation in AD γ-secretase results in the creation of an insoluble 42 amino acid peptide called Aβ42, or Aβ. Aβ peptide clusters come together to form aggregates known as β-amyloid plaques. The α-secretase enzyme cleaves APP at specific locations, which stops Aβ from being produced. Hence, it has a protective effect [124]. Aβ42 can induce the phosphorylation of APP at Thr668 through different kinases, when APP is phosphorylated it interacts with BACE1, enhancing Aβ production through increased APP cleavage by BACE1, Aβ monomers may associate together and form aggregates of different sizes, morphologies and leading to amyloid deposition in the brain inducing neuro-inflammation, oxidative damage, and calcium dys-homeostasis. It was found that higher concentrations of Aβ are toxic to neurons. Increasing synaptic Aβ, either through direct administration or by inhibiting its degradation, enhances synaptic release and neurotransmission by binding to APP and promoting its dimerization, which results in calcium influx and vesicle release [145].

Amyloid hypothesis, accumulation of Aβ in the brain is the primary influence driving AD pathogenesis. The rest of the disease process, including formation of neurofibrillary tangles containing tau protein, is proposed to result from an imbalance between Aβ production and Aβ clearance. “Aβ leads to disease an equally valid explanation is that mutation leads to disease leads to increased Aβ” as mentioned by Christian Behl which is an alternative perspective suggested that Aβ might stand at the end of the mutation-driven process rather than at the beginning neurodegeneration in AD. However, its relevance is questionable as some individuals with high amyloid remain cognitively normal, and several anti-Aβ medications have only minor advantages. Alternative models highlight neuro-inflammation, tau pathology, and mitochondrial failure [12].

### Neurofibrillary tangles (Tau hypothesis)

Abnormal bundles of filaments localized in neuronal axons, dendrites, and perikarya are known as neurofibrillary tangles (NFTs) (Ayaz et al. [9]). The two filaments are wrapped helically around one another, giving the ultrastructure of these filaments which has a regular constriction or straight appearance and hence their corresponding term paired helical filaments (PHFs). Further research reveals that PHFs have two c-shaped units in their core cross-section and overall resemble a twisted ribbon [77]. Another lineament of AD is the presence of neurofibrillary tangles (NFTs), which are aggregates of hyper-phosphorylated Tau protein released from microtubules, Tau protein normally keeps and stabilizes the neurons structure. Hyper-phosphorylation of Tau loses its ability to bind to microtubules and detaches, leading to microtubule destabilization and the aggregation of tau into NFTs. These tangles disrupt normal neuronal function and contribute to neurodegeneration [105]. Also, Aβ can induce Tau phosphorylation through specific kinases; Aβ also modulates Tau through the regulation of protein kinases and phosphatases. These returns suggest that Aβ initiates a pathway leading to Tau-dependent synaptic dysfunction. So, the Aβ and tau pathologies cause irreversible synaptic damage and neuronal dysfunction in brain regions responsible for learning and memory [144].

### The cholinergic hypothesis

The cholinergic hypothesis deducts that the cognitive decline observed in AD patients is due to reduced synthesis and activity of the acetylcholine (ACh) neurotransmitter in the brain [117]. The progressive decrease in the number of cholinergic neurons resulting from cholinergic neuronal death is another factor contributing to the pathophysiology of AD [27]. There is a critical link between cholinergic neuron deficits and cognitive impairments in AD patients also a marked reduction in the activity of choline acetyltransferase (an enzyme responsible for acetylcholine synthesis) was detected. Significant degeneration in the basal forebrain (a primary hub of cholinergic neurons), was observed. These observations, paired with the established role of acetylcholine in memory and learning processes, formed the basis of the cholinergic hypothesis [115]

The cholinergic hypothesis laid the groundwork for realizing AD and help in the creation of treatments such as acetylcholinesterase inhibitors (which responsible for the degradation of acetylcholine). Acetylcholine has been found to play an immunomodulatory role in addition to its role in neuron-to-neuron communication, influencing microglial activity and the broader inflammatory milieu within the AD brain. The cholinergic system works in interplay with other neurotransmitter systems, particularly the glutamatergic and GABAergic systems, and so any cholinergic deficits might disrupt this balance, leading to excitotoxicity and further neuronal damage [117]. ACh binds to presynaptic nicotinic receptors, causing the release of neurotransmitters from presynaptic neurons. Serotonin, acetylcholine, norepinephrine, and glutamate are among the neurotransmitters that play a crucial role in mood and memory and are all implicated in the pathophysiology of AD (Mir et al. [86]). Cognitive function steadily declines as a result of a gradual reduction in serotonin receptors and transporters in the AD brain [1]. Norepinephrine is also assumed to be the cause of the behavioral and psychological symptoms of dementia, in addition to memory loss [52].

Over 75% of neuronal loss has been reported in several brain areas at the later stages of AD. In the brain, acetylcholine (ACh) functions as a neurotransmitter that is involved in memory and impulse transmission. Impaired cognition is closely correlated with cholinergic neuron loss. AD causes a decline in norepinephrine levels as well as the death of neurons [49]. In AD, adrenaline, serotonin, and ACh neurotransmitters are produced at a reduced level in the cerebral cortex [2].

### Mitochondrial and oxidative stress hypothesis (role of mitochondria in reactive oxygen species)

Oxidative stress arises due to defect in equilibrium between the production of reactive oxygen species (ROS) and reactive nitrogen species (RNS), and the cell’s capacity to neutralize these reactive products or repair the damage caused by them. This imbalance is associated with mitochondrial dysfunction and can result in harming the cellular components such as lipids, proteins, polysaccharides, DNA and RNA [41]. Brain membrane phospholipids rich in polyunsaturated fatty acids are susceptible to free radical attacks, leading to lipid peroxidation that is an important feature in AD. The brain is highly susceptible to oxidative stress due to its energy need, high oxygen consumption, and elevated mitochondrial activity. Neuronal cells contain high levels of lipids and iron (that was found in Aβ and NFT deposits), catalyzing H_2_O_2_ into hydroxyl radical by the Fenton reaction. This reaction is an oxidation process in which iron salts (Fe^2+^) catalyze the oxidation of organic substrates with H_2_O_2_ result in production of highly reactive hydroxyl radicals which are potent inducers of oxidative stress. Also, this reaction forms a self-propagating loop in which the free radicals generated trigger oxidative damage to cellular components, promoting Aβ plaque formation and accelerating ROS production. Biometals such as iron, zinc, and copper play critical roles in Aβ aggregation and neurodegeneration, elevated levels of oxidative damage markers are found in individuals with symptoms of AD [126].

Mitochondria is the powerhouse of the cell, are crucial in oxidative phosphorylation and energy production. This process makes them a significant source of ROS. Massive amount of H_2_O_2_ originates from mitochondria as a result of the dis-mutation of superoxide anion produced during electron leakage. This makes mitochondria a major source of ROS contributing to oxidative stress that severely impacts proteins involved in mitochondrial ATP production and glycolysis. Mitochondrial dysfunction is obvious early in AD and affects mitochondrial function, including energy metabolism, calcium homeostasis, and the expression of mitochondrial DNA (mtDNA) [100]. Glucose metabolism, closely linked to cognitive function, is essential for determining AD progression. In AD, energy metabolism genes are decreased, the reduced glucose metabolism in the AD brain is associated with decreased activity of mitochondrial electron transport chain enzymes like pyruvate dehydrogenase, alpha-ketoglutarate dehydrogenase complex, and cytochrome oxidase. This reduction linked with clinical symptoms and is consistent with the presence of Aβ plaques. In addition, AD brains show high levels of mtDNA oxidation and mutations compared to age-matched controls, largely due to the closeness of mtDNA to ROS generation sites in the mitochondria. These mutations compromise mitochondrial function and reduce mitochondrial numbers in affected neurons. Therefore, excessive mitochondrial fission may enhance oxidative and nitrosative stress in AD pathology [23].

The brain tissues in AD patients undergo lipid peroxidation in reaction to Aβ, resulting in an accumulation of oxygen (ROS) and nitrogen (RNS)-reactive species. These reactive species interact with other molecules to become more stable. During this process, reactive species form molecular connections with other molecules, and free radicals, or high-energy electrons, are liberated. Because this is a lasting reaction, the reactive species permanently change the structure and function of certain biological molecules when they adhere to them [109]. This also resulted in genetic modifications. As the brain's high rate of oxygen consumption, high lipid content, and low level of antioxidant enzymes, make it particularly susceptible to the adverse consequences of oxidative stress. Numerous issues are brought on by this oxidation process in neurons, such as irreversible damage to DNA and an increase in pro-inflammatory cytokines [69]. Oxidative stress has an imperative role in the early stages of AD since it has been associated with the creation of amyloid plaques and NFTs [108].

### Inflammation hypothesis

Inflammatory response in the central nervous system (CNS) that fails to be resolved involves the activation of glial cells (the CNS innate immune cells) and the release of pro-inflammatory factors during neuro-inflammation. Stimulated microglia undergoes morphological changes and initiates a variety of responses. Aβ is a typical trigger for microglial activation. The activated glial cells migrate towards senile plaques, engulf Aβ, and release enzymes to break down Aβ but over prolonged periods; they might become less efficient at handling Aβ but continue to generate pro-inflammatory cytokines [144], so Aβ accumulation in AD patients' brains activates microglia and leads to pro-inflammatory pathways. Furthermore, active microglia phosphorylated tau proteins, triggering NFTs to propagate and accumulate in neurons [24]. Aβ also causes the formation and activation of the NLRP3 inflammasome within microglia, Interactions between microglia and tau protein in the later stages of AD may contribute to increased tau phosphorylation and exosomal tau secretion, thereby promoting the spread of tau. With the exaggerated activation, the complement cascade potentially leads to aberrant synapse pruning by microglia, further exacerbating AD pathology [144]. The presence of immune system cells and tau proteins in AD patients' brains suggests that the neuro-inflammatory process contributes to AD pathogenesis [51]. The hypothesis is reinforced by epidemiological data, which demonstrate a lower prevalence of AD among patients treated with anti-inflammatory medicines for chronic inflammatory disorders as arthritis [106]. Some studies suggest a reduced prevalence of AD with NSAID use, others show no benefit or even potential harm in those with established AD, according to certain observational meta-analyses of cohort studies, using NSAIDs may somewhat lower the risk of developing AD. On the other hand, naproxen and celecoxib did not stop Alzheimer's from starting, and they might have even had negative effects in the early stages, according to the ADAPT randomized controlled trial. AD cannot be effectively treated with NSAIDs in general. Additionally, they don't prevent AD from developing after prolonged use [7]. While inflammation is not the only factor leading to AD, it plays a role in the illness's progression [134].The hyper activation of microglia in Alzheimer's patients has been linked to polymorphic genes that generate Interleukin-1 (IL-1) and tumor necrosis factor- α (TNFα) [24]. Consequently, the production of potentially neurotoxic substances as chemokines, proteolytic enzymes, ROS, and cytokines causes the degeneration of neurons [146]. According to the inflammatory theory, Alzheimer's disease may be triggered by genetic variations that alter the inflammation process [147].

At the same time, mitochondrial dysfunction increases ROS, damaging cholinergic synapses and activating microglia, which in turn amplifies neuroinflammation. This vicious cycle between oxidative stress and inflammation accelerates neuronal death, indicating the need for multi-target strategies that support cholinergic function, protect mitochondria, and reduce inflammation [35]

## Pharmaceutical medications for Alzheimer's disease

Nowadays, anti-AD drugs pharmacologically work by increasing the amounts of ACh at brain synapses via the inhibition of acetylcholinesterase (AChE) and butyryl-cholinesterase (BChE) enzymes. They minimize the symptoms of the disease and reduce its progression rather than cure it completely [2]. Other medications address the symptoms of cognitive decline, and concomitant depression expressed by AD patients regretfully, there is now no effective therapeutics available to reduce or stop the brain's neurons malfunctioning and eventual death, Available pharmaceutical preparations include Tacrine (Cognex ®), Donepezil (Aricept®), and Galantamine (Razadyne®) to suppress cholinesterase and improve acetylcholine levels [57]. Donepezil acts also as anti-neuro-inflammatory through reduction of atypical glial cell activation (Moss et al. [89; 8]). Rivastigmine (Exelon®) is a selective Ach activity enhancer in the hippocampus and cerebral cortex that improves cognitive performance and a slowdown in the APP's production [62]. Other drugs act on glutamate-enhancing pathways such as memantine (Namenda), a NMDAR antagonist (De Sousa Rodrigues et al. [34]). Aducanumab (Aduhelm) diminishes soluble and insoluble Aβ aggregates through binding to AB epitopes [47]. A newly introduced formulation of memantine and donepezil in extended-release formula (Namzaric) has also proved effective [46]. The Food and Drug Administration (FDA) approved medications for AD, but they have serious side effects like weight loss, hepatotoxicity, diarrhea, gastrointestinal issues, nausea, and diminished efficacy (Ayaz et al. [9]).

Memantine is an antagonist of the N-methyl-D-aspartate receptor (NMDAR) subtype of glutamate receptor. Studies reveal a beneficial drug-drug interaction, indicating that the combination of Memantine and Donepezil could significantly increase the probability of five-year survival in AD patients and different doses of Memantine combined with Donepezil have beneficial potential in improving behavioral and psychological symptoms, cognitive function, and daily living ability of patients with moderate to severe AD [137].

In addition to that the majority of current pharmaceuticals minimize the symptoms of the disease other limitations of treatments for AD include penetrating the BBB that functions to selectively restrict the passage of hydrophilic medicines and most molecules, consequently, the transportation of medication to the brain require the utilization of techniques such as nano (specific lipophilic molecules of very small size that can diffuse) particles that possess the ability to transport these substances across the BBB. Lipidation of a medication has been explored as a means of facilitating its transport to the CNS [58].

Also, these drugs can only control or delay the development of the disease, cannot reverse or cure it completely due to the complexity of the disease, success rates remain low, in addition to having certain side effects, such as gastrointestinal adverse reactions, diarrhea, nausea and vomiting and other symptoms. However, the actual clinical application of drugs is still relatively limited, and the efficacy and safety of most drugs are still continuously being verified [135].

It is challenging to compare efficacy, safety, and evidence quality as most research evaluates them independently. For instance, donepezil has gastrointestinal adverse effects as nausea and diarrhea but only slightly improves cognitive. While curcumin and resveratrol exhibit antioxidant and anti-inflammatory benefits in small trials, they lack consistent large-scale clinical validation. Natural agents such as ginkgo, saffron, and Melissa officinalis have shown some cognitive benefits over a placebo in small randomized trials; however, high-quality, large-scale evidence remains limited. The efficacy and safety profiles of pharmaceutical and natural therapies have not been directly compared under the same clinical circumstances, despite the fact that both exhibit promise. The combined benefits of medicines and alternative treatments are also significantly superior to those of allopathic treatment alone. For the treatment of AD, several herbal therapies combined with FDA-approved medications are more promising and successful (Sheikh et al. [118]).

## Natural compounds as complementary or alternative therapeutic use in Alzheimer's treatment

Plenty of naturally occurring substances and the compounds derived from them have been extensively studied for their potential for improving cognition function in AD patients [9]. Compared to conventional pharmaceuticals, herbal therapies are believed to be less harmful and more successful in treating AD as they have already been demonstrated to be safe among the native populations [51]. Secondary metabolites are ubiquitous in plants, and some phytochemical such as alkaloids, flavonoids, lignins, polyphenols, sterols, triterpenes, and tannins have anti-cholinesterase and anti-amyloidogenic characteristics and have been shown to have therapeutic effects on memory retention (Md. Bhakshu et al. [81]). Phytophenolic chemical substances have a vital role and act as anti-inflammatory, antioxidants and inhibiting agents for a variety of pharmacological targets such as AchE, which presents in the hippocampus that reduces the concentration of ACh in the cerebrum. ACh levels returning throughout the brain has been used in recent decades to provide symptomatic therapy for AD (Md [81]).

### Curcuma longa

The ginger plant *Curcuma longa*, known as Curcuma, is a member of the *Zingiberaceae* family [21]. Pre-clinical studies on curcumin have shown its potential in preventing inflammatory pathways, lowering Aβ deposition, and enhancing cognitive function in Alzheimer's disease [111]. Curcuminoids are strong antioxidants that prevent the manufacturing and diffusion of free radicals. Curcumin can decrease brain water content, improve neurological impairment, lower mortality, and reduce infarct volume with a single dose (1 and 2 mg/kg, intravenously). Additionally, curcumin can increase heme oxygenase activity, which reduces the apparent appearance of β-amyloid plaques in animals with AD. The normal structure and function of brain arteries, mitochondria, and synapses remain intact by curcumin, which also minimizes the risk factors for chronic diseases and AD [21]. Limiting use of curcumin like most natural compounds include low bio-availability, decreased solubility, limited BBB permeability and rabid meatbolism [44].

### Withania somnifera

As a member of the nightshade (*Solanaceae*) family, ashwagandha has immune system preservation, free radical scavenging, and antioxidant properties [84]. By reducing apoptotic cell death, restoring synaptic function, boosting antioxidant effects, and inhibiting nuclear factor-B activation and β-amyloid production, it assists in treating AD. The anti-inflammatory, anxiolytic, anti-apoptotic, and neurite-promoting features of ashwagandha root may cause its positive effects. Furthermore, it boosts antioxidant defenses, restores energy levels, and relieves mitochondrial dysfunction. Ashwagandha can penetrate the blood–brain barrier and reduce inflammation in the brain. Generally it is safe for chronic administration except for the possibility of nausea or diarrhea incidence [70].

### Convolvulus pluricaulis

*Convolvulus*, an Ayurvedic plant a part of the *Convolvulaceae* family, has been stated to enhance learning and memory. It declines phospholipids and cholesterol, raises the concentration of proteins in the brain, and relieves psychological, chemical, and traumatic stress. A variety of pharmacological activities are exhibited by the plant's phytochemicals, such as nootropic and memory-enhancing effects [82].

### Centella asiatica

*Centella asiatica*, commonly called Asiatic pennywort or gotu kola, is a widely used plant in Asian nations. Research reveals that it enhances cognitive function, declines β-amyloid levels, and protects against brain injury. Additionally, studies on animals have shown that it has antistress, antidepressant, anxiolytic, and seizure-preventing effects. Asiaticoside and Asiatic acid have been shown in animal studies to have neuroprotective, anti-depressive, and anxiolytic properties. It has also been demonstrated to enhance executive function memory, declining hippocampal mitochondrial dysfunction in rats with elevated β-amyloid levels and also activates the antioxidative defense system [107].

### Celastrus paniculatus

The medicinal herb Celastrus, a member of the *Celastraceae* family, has been shown to possess neuroprotective properties. According to research, it protects rats from kainic acid-induced neurotoxicity, inhibits NMDA receptor-driven whole cell currents, and ceases glutamate-induced neuronal damage. Additionally, it improves spatial navigational memory deficits caused by scopolamine, a muscarinic cholinergic receptor antagonist [13].

### Coriandrum sativum

A member of the *Apiaceae* family, *Coriandrum sativum* is an annual herb that has been shown to have anti-inflammatory, cholesterol-lowering, and antioxidant effects that enhance cognitive function in male Wistar rats. In Iranian folk medicine, it has been utilized for relieving sleeplessness and anxiety. According to animal models, fresh plant extracts exceed dry extracts in reducing immobility time. Additionally, the hydro alcoholic extract of C. sativum suppresses the development of dark neurons and apoptotic cells in the hippocampus and decreases burst discharges [102].

### Ficus carica

*Ficus carica*, also known as the common fig, fig tree, and its fruit has been indicated to have minor memory-enhancing properties at low doses. Its antioxidant effects are especially due to quercetin, which plays a role in memory loss and AD. The effectiveness of Ficus carica on cognitive functions was assessed in a study including mice with memory impairments and normal mice. The fruit parts exhibited significant bioactivity against ROS radicals [122].

### Magnolia officinalis

A* Magnoliaceae* family member has been shown to help in treating memory problems. Its antioxidant properties, which include honokiol and magnolol, have been shown to help treat (AD). The plant's ethanolic extract is especially potent. Scientific studies on the biological activity of magnolol and honokiol show that, although they are optical isomers, they exhibit slightly different in vitro and in vivo activities. Magnolol, for instance, can increase apoptosis and inhibit the growth of cancer cells in esophageal, osteosarcoma, colorectal, and other cancers [22, 26]. Furthermore, Magnolol can reduce oxidative stress in adipose tissue [97] and neuro-inflammation and stress in mice with prefrontal cortex [28].

### Myristica fragrans

A member of the *Myristicaceae* family has been shown to treat many conditions including AD. A study has demonstrated the plant's antibacterial, antioxidant, and antidepressant features [123].

### Bacopa monnieri

It is a creeping perennial herb belonging to the *Scrophulariaceae* family, used by traditional healers to treat AD and memory loss. It is an adaptogenic, neuroprotective, antibacterial, and memory enhancer that contains sterols, saponins, and alkaloids. According to studies, *bacopa monnieri* helps AD patients think more effectively by enhancing cognition, minimizing cholinergic degeneration, and improving their cognitive functioning [128].

## Limitations of using natural products in Alzheimer's

Natural compounds have shown potential in preclinical and clinical studies for AD therapy. Still, their effectiveness in clinical trials has been limited due to absorption, bio-distribution, metabolism, bioavailability, and clearance. Low bioavailability is a major limitation, as these compounds can be prematurely cleared from blood circulation due to low stability to light, oxygen, and temperature. Additionally, low oral bioavailability hinders the efficient accumulation of required doses in brain tissue for therapeutic effects. Their nano delivery can solve the previously mentioned problems. By enhancing the pharmaceutical aspects of natural compounds, to be able to overcome these limitations and boost their therapeutic efficacy [32].

## Using nanoparticles in nanomedicine; a potential approach to drug delivery across the blood brain barrier

Nanotechnology has several applications because of its interdisciplinary character and ties to physics, chemistry, biology, electronics, biomedicine, and materials science [78]. Its formal base is the synthesis, characterization, manufacture, and use of nanoscale materials (1–100 nm). Applications for nanoparticles (NPs) can be found in medicinal research, manufacturing, and diagnostics. When appropriately formulated, they can function as imaging agents, delivery vehicles for proteins, nucleic acids, and encapsulated medications, and enhance detection selectivity and sensitivity.

NPs have excellent stability, high drug bioavailability, high loading capacity, and good biocompatibility. NPs can have ligands like proteins, antibodies, or nucleic acids attached to their surface to target certain cells or organs. The previously mentioned features hold promise for gene therapy, tissue regeneration, and imaging developing biomarkers for the detection of different illnesses and cells, and medication delivery [75].

When administered into the body, NPs be faced with some challenges that can compromise their efficacy and safety. Stability; once administered, NPs must remain stable in the bloodstream for long time enough to reach their target site. Physical instability; NPs aggregation that cause particle clumping and sedimentation. Biological instability caused by the adsorption of biomolecules including proteins, lipids, and enzymes, onto their surface, this adsorption can alter the surface properties of nanoparticles, modifying their interactions with cells and leading to rapid clearance by the phagocytes. The biological instability of nanoparticles could also be attributed to enzymatic degradation that affects the structural integrity of NPs leading to a reduction in their therapeutic effect. Less bio-compatibility resulted in toxicity following administration [74].

Nanoparticle systems can be administered via a variety of techniques including oral [56], nasal [76]. Nose-to-brain drug delivery is novel approach that connecting the nasal cavity to the brain, this method bypasses the BBB, enabling direct and efficient transport of therapeutic agents to the CNS, intranasal (IN) administration has several advantages over the traditional drug delivery routes through passing the gastrointestinal tract and liver metabolism, lowering the risk of drug degradation during first-pass metabolism, improving bioavailability. Furthermore, IN delivery help rapid drug absorption and onset of action, making it beneficial in conditions requiring immediate therapeutic effects. The localized administration also minimizes systemic side effects, as the drug is directed specifically to the brain. Additionally, the ease of self-administration makes IN delivery a patient-friendly, enhancing compliance and accessibility for chronic or acute treatments (Nguyen et al. [93]). Also NPs can be administered via parenteral [132], intramuscular (Narain [92]), and subcutaneous [94]. The majority of nanoparticle systems employed are made up of polymers that are synthetic or natural, proteins, and polysaccharides that contain the therapeutic ingredient for targeted brain delivery [53].These polymeric nanoparticles exhibit promising brain-targeting properties as well as the ability to modify the drug-releasing pattern for controlled, sustained, or extended drug effects. They also shield the drug in situ by interfering with its lipophilic requirement to pass the BBB, which can also shield the drug from the effects of first pass metabolism or enzymatic degradation [40]. These properties can help to increase bioavailability, lower the necessary therapeutic dose, and ultimately reduce drug toxicity and systemic adverse effects.

To address these problems may face NPs, an effective approach is to modify the NP surface, changing their physicochemical properties and bioactivity, which is the surface functionalization of the NP with polymers such as polyethylene glycol (PEG), that improves stability by preventing aggregation and protein adsorption, also maintaining consistent size and prolonging circulation time in biological environments. This process extends the residence time of nanoparticles at the target site, additionally enhance drug absorption. Additionally, surface modifications can alleviate toxicity by using biocompatible coatings that cover the NPs from causing immune responses and minimize interactions with non-target cells. NPs can be conjugated with ligands, such as antibodies, peptides, or small molecules that recognize and bind to specific receptors on the cell membrane and facilitating uptake by target cells and enabling controlled drug release. This targeted approach, known as active coating, enhances the accumulation of nanoparticles in diseased tissues while reducing off-target effects and systemic toxicity that may be helpful in reaching brain and treating difficult disease including AD [74].

A variety of nano-systems can be found, including NP, nano-micelles, nano-spheres, nano-gels, nano-suspensions, and nano-liposomes. The selection of an appropriate type of nano-system is contingent upon the characteristics of the targeted cell or region, the drug's nature and mass, the necessary drug release pattern, the type of dosage form, and, ultimately, the intent of the drug challenge to enter the brain or central nervous system [73]. To enhance transport and shield it from deterioration, the appropriate nanoparticle type (lipid, polymeric, or hybrid) must be chosen based on the characteristics of the phytochemical (e.g., stability, solubility). However, there are obstacles to bringing these systems to clinical settings, such as challenges with large-scale production, ambiguous regulatory frameworks, and worries about long-term safety because nanoparticles can deposit in organs [59]. Nano suspensions and nanocrystals have emerged as viable solutions to the problems associated with poorly water-soluble medication formulation. Additionally, they offer a lesser-known but adaptable platform with a variety of therapeutic uses. They may be included in a wide range of drug delivery forms, such as hydrogels, tablets, microneedles, micro-particles, and even liposomes with functionalized areas [106].

One of the basic challenges faced by the pharmaceutical industry is the poor water solubility and insufficient bioavailability of drugs that cause patients need to consume higher drug dosages to achieve the desired therapeutic effects, leading to many health problems, to overcome these issues, the development of different nanomedicine delivery systems may be helpful getting the desired Pharmacokinetic profile [72].

Some delivery systems include biodegradable poly (lactic-co-glycolic acid) (PLGA- NP) have been used in delivering drugs to target tissues due to their excellent biocompatibility, PLGA-conjugated drugs can attenuate pathology of AD, also native PLGA can suppress Aβ aggregation and protect neurons against Aβ-mediated toxicity, PLGA degrades over time through hydrolysis and release the encapsulated drug. Biodegradability, tunable drug release profiles and ability to encapsulate both hydrophilic and hydrophobic drugs PLGA all are very helpful advantages; however degradation products may have acidic effects in some cases [133].

Nano-spheres or matrix-type are polymeric NPs composed of spherical polymeric matrices that act as carriers of therapeutic drugs. The biologically active agents are spread uniformly across the polymeric core and are released by the diffusion mechanism. Besides, active agents can be adsorbed on the surface of nano-spheres. These nanostructures have maintained and controllable drug-release profiles and high loading capability for less water soluble drugs so they have the potential to integrate hydrophobic drugs and can offer sustained drug release delivering drugs to the brain that may be helpful in the treatment of AD, but some materials may require specialized preparation for drug loading [55].

On the other side, nano-liposomes are microscopic vesicles containing a phospholipid bilayer. They facilitate the delivery of hydrophilic and lipophilic drugs due to the amphipathic properties of their constituent elements. Advantages including targeted delivery to the brain, low toxicity, biodegradability and lack of immunogenicity have made liposomes a very suitable carrier in drug delivery. These capsules can carry drugs to different parts of the body by encapsulating drugs inside them. Delivering Aβ antibodies or other therapeutic drugs to reduce amyloid plaques in the brain help AD treatment [103].

## Benefits of utilizing nanoparticles for drug delivery targeting the central nervous system

Nanoparticles tend to provide an abundance of noteworthy benefits that might be utilized in the medical domain to create novel and precise dosage forms. The advantages of using nanoparticles for parenteral administration include their high surface area-to-volume ratio, high drug-loading capacity, reduced potential for chemical interactions or toxicity, ease of manipulation due to their small size and surface characteristics, ability to be used for both passive and active drug-targeting strategies, sustained monitored release for drug production, and specific to the site targeting through the use of magnetic guidance or attaching targeting ligands to the particle surface [101]. The required nanoparticle size, targeting properties, lipid or water solubility and their corresponding hydrophilic properties, both physical and chemical rigidity, surface electrical charge and permeability, biocompatibility, biodegradability, cytotoxicity, drug release profile, and antigenicity of the finished product are just a few of the many variables that influence the process of choosing the materials for nanoparticle manufacturing [140]. The drug's delivery design is challenged by blood brain barriers. Pharmaceutical dosage forms typically have relatively low bioavailability and are unable to readily cross the BBB instead, they can only do so through active efflux or carrier-mediated transport in typical drug formulations [67]. Research carried out in vivo and in vitro verified that nanoparticles may pass across the BBB. They supported the delivery of nano-therapeutic materials into the brain, which implies that nanoparticles may be utilized for diagnostic purposes in addition to gene therapy and brain-targeted therapies [64].

## The optimal characteristics of nanoparticles for the transport of drugs to the brain:

1. Nontoxic, biodegradable, and biocompatible nanoparticles such as polylactide homopolymers and polylactide-co-glycolide polymer nanoparticles must be present [68].
2. The ideal size for a nanoparticle is less than 100 nm since larger particles have an increased rate of clearance, which impacts both biodistribution and bioavailability [48].
3. Certain types of nanoparticles were thought to be hazardous because of their physical as opposed to chemical surface characteristics, physical stability and avoiding aggregation in the circulation are essential [6].
4. Extended blood circulation duration, whereas just PE-Gylated nanoparticles exhibited reduced absorption and extended duration [29].
5. Non-invasive gene-targeting brain delivery: PE-Gylated immuno-liposomes, for example, access the brain tissues without damaging the BBB through receptor-mediated transcytosis [39].
6. Research on how cost-effectively nanomedicine could be used in clinical settings should be conducted. The number of patients who can benefit from those treatments will be reduced as treatment costs rise [33].

## The therapeutic effects of nanoparticles derived from natural products on Alzheimer disease

### Curcumin

It has been demonstrated that curcumin-loaded nanoparticles (NPs) enhance curcumin delivery to the brain, reducing Aβ aggregates and enhancing memory and spatial learning in AD models [116]. Curcumin-loaded NPs administered intranasal this route has many advantages as mentioned in details including the NPs and IN pros have also demonstrated encouraging outcomes; following intranasal administration of 160 μg/kg, curcumin was found in plasma concentrations and brain levels. This implies that the olfactory and trigeminal pathways may deliver curcumin to the brain [130].

Regarding the mechanisms of curcumin-loaded NPs in ameliorating AD symptoms, it operates through several mechanisms: by inhibiting many inflammatory pathways like NF-κB, repress the production of pro-inflammatory agents; acting as a potent antioxidant, neutralizing free radicals and support the endogenous antioxidant enzymes to shield neurons from oxidative harm; disrupting Aβ plaque aggregation, a hallmark of AD, thus preventing their accumulation and promoting plaque clearance; and conferring neuroprotection by modulating cell survival pathways, such as PI3K/Akt, and inhibiting apoptotic processes [99].

### Quercetin

The flavonoid quercetin, present in fruits and vegetables, has many health advantages, such as antioxidant, neuroprotective, and cardio protective properties. It has been revealed to enhance neurogenesis in AD and prevent tau hyper-phosphorylation and Aβ aggregation. Nevertheless, its clinical use has been restricted due to its instability, poor permeability, and low solubility. NPs can prevent quercetin from degrading, increasing its brain bioavailability. In animal models of AD, quercetin-loaded NPs have been shown in vivo experiments to have strong antioxidant and anti-amyloid properties, which improve learning and memory impairments [85].

PLGA is a biodegradable polymer that has high stability, biocompatibility, and bio-degradability; it is usually degraded into individual monomers by hydrolysis of its ester linkages and then metabolized and removed by natural functioning of the cell. The intention of using PLGA-quercetin NPs is to overcome the aforementioned issues; increase the bioavailability within the cell as well as slow down release of quercetin into the blood stream, thereby enhancing its overall therapeutic activity [136].

### Resveratrol

Polyphenol resveratrol, which exists in red wine, peanuts, pomegranates, berries, and grapes, has been demonstrated to possess anti-inflammatory, anti-aging, anti-cancer, and antioxidant properties. It has been shown in Alzheimer's disease models to shield cells from the accumulation of Aβ plaque and tau protein hyperphosphorylation [98]. It has immunomodulatory potential through altering the cytokine production profiles of various glial cells. it intracellularly activates sirtuin 1 which is a signaling protein that normally decreases pro-inflammatory cytokines production including (IL-1, IL-6 & TNF-α) [44].

No cytotoxic or antioxidant effects were observed when resveratrol was coated onto solid lipid or selenium nanoparticles. However, the liver and intestines quickly metabolize free resveratrol, reducing its pharmacological advantages. The biocompatibility and long-term circulation of resveratrol were enhanced by nanostructured lipid carriers [110]. Limiting use of resveratrol like most natural compounds include low bio-availability, decreased solubility, limited BBB permeability and rabid meatbolism [44].

### Phytol

Diterpene phytol, which is a component of chlorophyll, possesses anti-inflammatory and anti-carcinogenic features [16, 19]. PLGA NPs loaded with phytol have been demonstrated to have anti-cholinesterase activity, prevent Aβ aggregation, and shield Neuro2a cells from Aβ toxicity [114]. The administration of phytol-loaded PLGA NPs to a transgenic Caenorhabditis elegans AD model was demonstrated to improve lifespan, inhibit chemotaxis behavior defects, and reduce intracellular ROS production [113]. The cognitive impairments in spatial and short-term memory caused by scopolamine were also alleviated in Wistar rats after 14 days of oral administration of phytol-loaded PLGA NPs (100 and 200 mg/kg) [112]. Phytol-loaded PLGA NPs have a strong ability to penetrate the blood–brain barrier in vivo, according to the study [131].

### Thymoquinone

Thymoquinone, a monoterpene with anti-inflammatory, antioxidant, and antimicrobial qualities, is found in *Nigella sativa* seeds. Neuroprotective effects have been demonstrated, especially in models of AD. However, because of its high lipophilicity, its bioavailability in the brain is restricted. Thymoquinone-loaded nanoparticle therapy can improve delivery and lower oxidative stress in specific regions of the brain, which helps to enhance neuroinflammation and neurodegeneration alterations in AD models. Additionally, streptozotocin-induced AD mice's brain protein aggregates declined by treatment with polysorbate-80 coated PLGA thymoquinone NPs, which effectively crossed the blood–brain barrier [141]. To gain thymoquinone helpful effects including regulation of anti-inflammatory reactions, antioxidant effects and regulating the neuro-inflammatory reactions caused by Aβ accumulations and to improve its therapeutic potential, which is limited due to the poor solubility, low absorption, and rapid clearance, nanotechnology offers a promising approach to enhance drug efficacy by improving targeted delivery and bioavailability. Solid lipid nanoparticles (SLNs) have gained significant attention due to their biocompatibility, biodegradability, high bioavailability, low toxicity, good storage capability, sustained release, and scalability (Ramachandran et al., [104]).

### Ginsenoside

The pharmacological effects of ginsenoside, which is an active component from Ginseng that is extracted from the roots of Panax ginseng Meyer, have been utilized in both traditional medicine and dietary supplements. Ginsenosides have been demonstrated to enhance neurodegenerative, anti-inflammatory, antioxidant, anti-diabetic, anti-aging, anti-depression, and immune-modulatory processes [54].

Nevertheless, there aren't many studies that use ginsenoside nano-formulation to treat AD. According to research conducted in vitro, encapsulated ginsenoside Rg3 dramatically reduces the formation of Aβ fibrils [131].

### Huperzine

The alkaloid Huperzine A, a powerful inhibitor of AChE, was extracted from the club moss Huperzia serrata [127]. Cognitive function and the ability to switch between tasks were significantly improved after taking 0.2 mg orally twice a day for 8 weeks [45]. A study by [83, 119] documented the effects of huperzine A complex administered intranasally on the brain. PLGA-NPs and huperzine A-loaded mucoadhesive were the names of the complex, which was externally adjusted by lactoferrin-conjugated N-trimethylated chitosan (Lf-TMC NPs). By promoting the distribution of huperzine A in the brain, the remarkable findings indicate a potential nose-to-brain NP delivery pathway for AD treatment [131].

### Andrographolide

Andrographolide (AG), a significant diterpenoid found in *Andrographis paniculata*, has pharmacological properties that include antiretroviral, antibacterial, antimalarial, anti-inflammatory, and antioxidant properties [142]. After being administered intravenously to the TgCRND8 AD mouse model, andrographolide loaded onto human albumin NPs was demonstrated to penetrate the brain parenchyma and cross the blood–brain barrier [15]. Additionally, giving andrographolide-loaded NPs intraperitoneally to AD mice for four weeks lessened astrocyte reaction and improved cognitive dysfunctions, demonstrating the anti-inflammatory nature of andrographolide. As andrographolide NPs can across the BBB, it can suppress the activation of astrocytes, which become overactive in AD and lead to neuro-inflammation. It was found that AG prevented Aβ oligomer damage, reduced Aβ and tau phosphorylation in mice, controlled amyloid plaque development, improve learning abilities in animal models of AD, enhance the cognitive function and restored spatial memory in mice, a model for AD nanoparticles could be a safe, effective, and non-invasive way to treat memory loss [95].

### Hesperetin

Citrus flavonoids like hesperetin, abundant in orange and grape juice, have been demonstrated to increase oxidative stress, neuro-inflammation, apoptosis, and memory impairment in the central nervous system [65, 90]. As with other phytochemicals previously discussed, hesperetin's clinical applications have been restricted by its rapid clearance from the body and low bioavailability due to its water insolubility [50]. In the AD model, treatment with hesperetin nanosuspension made by evaporative precipitation demonstrated a stronger neuroprotective effect than free hesperetin. Following treatment with nano-hesperetin at doses of 10 and 20 mg/kg orally for streptozotocin-induced AD rats, [50, 88]. Improvements in memory retrieval and recognition memory consolidation, increased antioxidant enzymes activity, and decreased lipid peroxidation in the hippocampus were demonstrated.

#### Naringenin

One flavonoid that is broadly found in citrus fruits is naringenin. According to reports, it has anti-inflammatory, anti-cancer, anti-hypertensive, anti-thrombotic, anti-atherosclerosis, antidiabetic, and antioxidant properties [143]. Naringenin has been shown to lower Aβ levels and shield cells from the neurotoxicity caused by Aβ1-42 [5]. Naringenin treatment also reduces neuro-inflammation and cognitive impairment caused by lipopolysaccharide [63]. Naringenin nanoemulsion decreased cellular ROS production and APP, BACE, and tau phosphorylation, it also significantly reduced the neurotoxic effects of Aβ [80].

Both hesperetin and naringenin NPs share many important protective roles in AD as they have antioxidant activity through scavenging the ROS and reducing their effect on biomolecules, reduce neuro-inflammation may affect the brain by modulating the inflammatory pathways and reduce the inflammatory effect due to the accumulation of Aβ [79, 14].

## Therapeutic challenges confronting nanomedicine

The recent utilization of nanotechnology in biomedicine has given scientists hope for the future study and treatment of neurological disorders, however, there are some obstacles involved in their application. Safety issues are raised by neurotoxicity caused by nano-delivery technologies in addition to the BBB, which is a significant obstacle to treatments [120].The emergence of oxidative stress is a frequent indicator of this neurotoxicity, which is primarily dependent on the shape, size, surface area, solubility, concentration, duration, and route of administration of the nanotherapeutic agent [66]. Both size and concentration affect the neurotoxicity of nanoparticles. For instance, in AD cell and animal models, particles smaller than 100 nm at concentrations greater than 50–100 µg/mL have been demonstrated to cause oxidative stress, mitochondrial malfunction, and neuronal death [66]. Despite all of this, the physicochemical features of NPs, as previously stated, make them promising candidates in nanomedicine. To address some of these issues, NP formulations must include biocompatible components that are biodegradable and easily eliminated from the system [125].The capacity for crossing the BBB is described more thoroughly in the next section. Likewise, the toxicities demonstrated are frequently dependent on the type of NP utilized, with surface functionalization providing a step forward in decreasing unwanted effects and interactions. Therefore, when it comes to selecting an NP and using it, there is not a "one-fits-all" strategy. Targeted therapies will be critical in the treatment of AD because cell-specific targeting is necessary to repair damaged or mutant genes without compromising the integrity of normally functioning genes and cells. However, further research on NPs is necessary before developing CNS medication. There is nowadays a lack of awareness of NP neurotoxicity, indicating a pressing need for more in vitro and in vivo investigations to offer a basis for future research. The development of an ideal nanoparticle might be aided by the use of developing technology, particularly in silico investigations, computer and mathematical modeling, and increased bioinformatics understanding crossing the BBB. As a dynamic barrier, the BBB controls the flow of biomolecules from the bloodstream into the brain, protecting the brain from bigger medicines and harmful substances. Even though this function is quite advantageous, it stands in the way of modern therapies (Cenã and Játiva [20]). NPs can cross this BBB because they are tiny molecules; most of them are less than 200 nm. In addition to size, other characteristics that give NPs the ability to cross the BBB include charge, particularly a positive charge, appropriate surface functionalization, and incorporation of targeting ligands like polyethylene glycol and cell-penetrating peptides for longer circulation times in vivo [20]. It has been shown, nonetheless, that as BBB permeability varies from rodents to human beings, selecting the appropriate in vivo disease model is vital to assessing the capacity of the NPs to traverse the BBB [20]. The predictive validity of AD treatments is significantly affected by host species variations in the structure and transport characteristics of the BBB. Compared to human BBBs, rodent BBBs which are frequently employed in preclinical research tend to be more permeable and show varying degrees of expression of tight junctions and efflux transporters, which could result in an overestimation of therapeutic brain delivery. Accordingly, although rodent models are helpful for understanding mechanisms, they are not very applicable to humans, and more sophisticated human-relevant models such as BBB chips are required for higher prediction accuracy [11]. Through in-depth study of transport molecules, scientists can develop treatments that make use of the body's built-in physiological barriers to deliver pharmacologically active substances to the brain securely and effectively. Regarding one implying a low molecular weight, non-ionization, the existence of hydrogen bonds [37, 96] and lipophilicity were the ideal characteristics for a nanocomposite to be able to cross the BBB [91]. So from the aforementioned points the mitigation strategies may include selection of the nano-material, size and optimizing the surface properties.

### Challenges in translating herbal neuro-therapeutics to clinical practice

There is lack of scientific index to evaluate safety and efficacy of herbal drugs. The quality of the trial drug must be tested for (batch-to-batch) matching the active components. It is very difficult to have active and control groups with the same color, smell and taste of the herbal drug. These challenges can be reduced by applying most recent methodologies and guidelines for clinical trials. Since the quality control of herbal medicines is sophisticated, relevant and appropriate requirements have to be established for the assessment of safety and efficacy for different categorized herbal medicines to reduce cost and expenditure, more efforts should be made for the integration of traditional medicine into national healthcare systems.

## Conclusion

Developing new effective treatments for neurodegenerative disorders like AD is interesting but not easy. Nanomedicine is a potent and effective tool help overcoming obstacles and challenges may appear during usage of traditional treatment including low-bioavailability, low solubility and targeting brain. The variety of commercial nanoparticles should be carefully tested for stability, toxicity, target delivery and their transport of drugs to the CNS must be maximized which is the role of new researches. Although nanomedicine has enormous promise for treating AD, specific next steps are required to take the idea from aspiration to reality. Addressing regulatory frameworks to guarantee safety and uniformity, developing scalable and repeatable nanoparticle manufacturing, and incorporating biomarker-guided targeting techniques to customize therapy and enhance effectiveness in clinical settings are among the top priorities.

## Supplementary Information

Below is the link to the electronic supplementary material.

Supplementary Material 1

## Data Availability

No datasets were generated or analysed during the current study.
