# Supplementary material for: Phytochemical based on nanoparticles for neurodegenerative alzheimer disease management: Update review
Source: Discov Nano. 2025 Oct 9;20(1):176. doi: 10.1186/s11671-025-04356-x (PMC12508342; doi:10.1186/s11671-025-04356-x)
Supplement: Supplementary file 1 — Supplementary Material 1 [file 11671_2025_4356_MOESM1_ESM.docx]

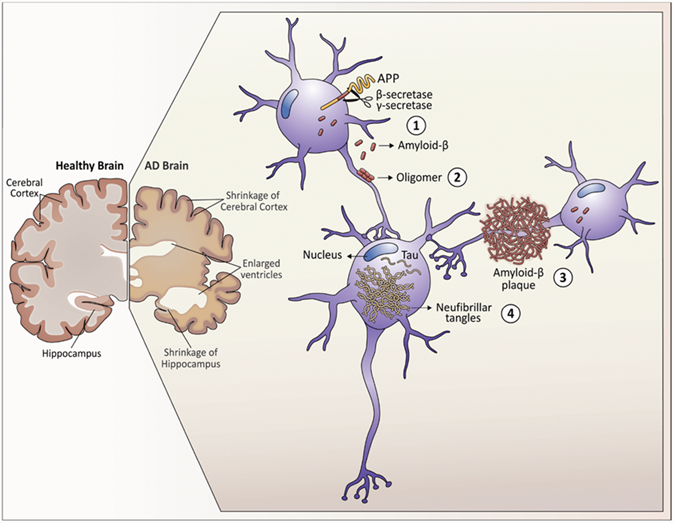


**Figure 1: the halmarkers amyloid-beta plaques and neurofibrillary tangles tau protein that characterized Alzheimer disease (Gomez et al. 2020)**


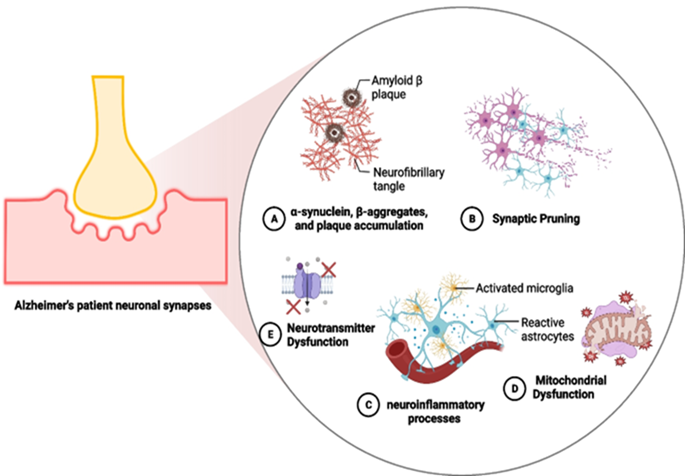


**Figure 2*:* Diverse mechanisms of Alzheimer's disease (AD*) (Moawad et al. 2024).***


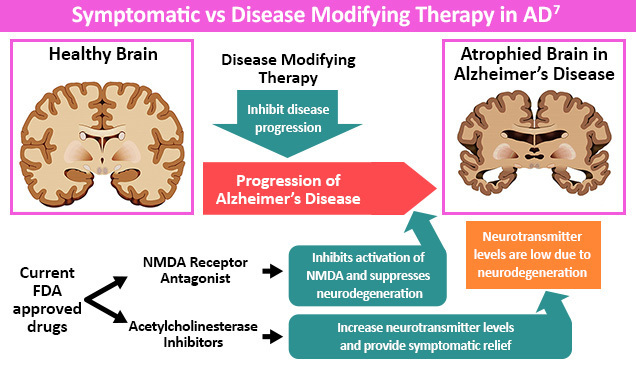


**Figure 3: current symptomatic vs disease modifying therapy in Alzheimer disease (Abeysinghe et al .2020)**


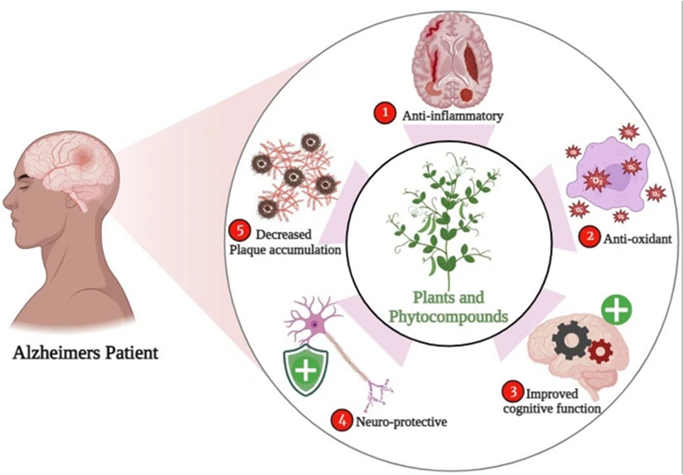


**Figure 4: vital role of plants and phytochemicals on Alzheimer disease**

**.( Bordoloi et al. 2024)**
